# Lipid-Lowering Effect of the *Pleurotus eryngii* (King Oyster Mushroom) Polysaccharide from Solid-State Fermentation on Both Macrophage-Derived Foam Cells and Zebrafish Models

**DOI:** 10.3390/polym10050492

**Published:** 2018-05-03

**Authors:** Hua Wei, Shang Yue, Shizhu Zhang, Ling Lu

**Affiliations:** Jiangsu Key Laboratory for Microbes and Functional Genomics, Jiangsu Engineering and Technology Research Center for Industrialization of Microbial Resources, College of Life Sciences, Nanjing Normal University, Nanjing 210023, China; weihua@njnu.edu.cn (H.W.); yueshang3639@163.com (S.Y.)

**Keywords:** lipid-lowering, *Pleurotus eryngii* (king oyster mushroom), polysaccharide, solid-state fermentation, zebrafish

## Abstract

Hyperlipidemia is a key risk factor in inducing fatty liver, hypertension, atherosclerosis and cerebrovascular diseases. Previous studies have verified that polysaccharides from fruiting bodies (PEPE) of *Pleurotus eryngii* (king oyster mushroom) are capable of decreasing the lipid content. In this study, the *P. eryngii* polysaccharide is obtained by solid-state fermentation (PESF) using lignocellulosic wastes, corn-cobs and wheat bran. The high-performance liquid chromatography (HPLC) assays indicate that PESF has a similar composition to that of PEPE. Meanwhile, PESF has no detectable toxicity and is able to significantly inhibit foam-cell formation in murine macrophage cells (RAW264.7) induced by oxidized low-density lipoprotein. Further verification indicates that PESF has lipid-lowering effects during the lipid absorption phase in a zebrafish hyperlipidemia model. Our findings suggest that the *P. eryngii* polysaccharide from solid-state fermentation (PESF) can be used as a valuable lipid-lowering food additive or raw materials for producing lipid-lowering drugs.

## 1. Introduction

Hyperlipidemia is the most common form of dyslipidemia and is a key risk factor in inducing fatty liver, hypertension, atherosclerosis and cerebrovascular disease. The current clinically used hypolipidemic drugs, such as statins, fibrates, and niacin, often cause undesirable adverse effects including myopathy, rhab-domyolysis, and polyneuropathy, which still need to be overcome [1]. Therefore, the interest in potential natural lipid-lowering compounds obtained from edible mushrooms has increased in recent years. 

Edible and medicinal mushrooms have a long history of use in folk medicines and health foods in most Asian countries [2]. For decades, extensive studies have revealed that these fungi have many specific bioactive compounds with anti-oxidant, anti-tumor, hypoglycaemic, hypolipidemic, and immune-stimulating activities. Many lines of evidence have identified mushrooms’ (or their components’) own important natural agents for the control of hyperlipidemia due to their contents of polysaccharides, dietary fibre, lovastatin and some other particular compounds. Interestingly, our previous studies have indicated that the natural polysaccharides from edible and medicinal mushroom fruiting bodies and submersed fermentation have bioactive elements that combat hyperlipidemia in cell-line or mouse models [3,4,5].

However, whether the bioactive polysaccharides can be produced using lignocellulosic wastes from mycelia solid-state fermentation (MSSF) remains uncertain. The production of fungal biomass by MSSF has obvious advantages including the low cost of equipment, power and especially raw materials, which are generally composed of a mix of lignocellulolitic wastes.

To date, most studies with hyperlipidemia models are focused on mammals, including mouse, rat, rabbit and hamster models of hyperlipidemia induced by high-fat diets. However, these models are often time-consuming, labor-intensive and expensive [6,7,8]. Drug screening with cell models is efficient but these models lack organ structures. With advantages such as transparency of larvae, short reproductive cycles and abundant generation, the zebrafish is emerging as a vertebrate animal model for in vivo assessments of drug efficacy and toxicity [9]. Furthermore, the morphological and molecular basis of tissues and organs in the zebrafish is either identical or similar to humans, respectively. The sequences and the presumed functions of many genes that are important in vertebrates are conserved in the zebrafish [10]. Meanwhile, some investigators have assessed global lipid stores in the zebrafish [11,12,13]. Therefore, the zebrafish can be developed as an effective animal model for the study of body lipid metabolism [14].

The objective of this study is to determine whether the *P. eryngii* (king oyster mushroom) polysaccharide with lipid-lowering effects can be produced by mycelia solid-state fermentation using corn-cobs and wheat bran as fermented materials. We developed a zebrafish hyperlipidemia model with the advantages of a short duration requirement for an in vivo efficacy assessment and research on lipid-lowering food additives or drugs. 

## 2. Materials and Methods

### 2.1. Raw Materials and Preparation 

The oxidized low density lipoprotein (oxLDL) was purchased from Yiyuan Biotechnology Co., Ltd. (Guangzhou, China). DMEM medium, fetal bovine serum (FBS), penicillin, and streptomycin were purchased from Wisent Biological (Naning, China) Co., Ltd. Egg yolk power was purchased from Tianyuan Company of Beijing.

All other un-described reagents used in this study were of analytical grade. 

### 2.2. P. eryngii Mycelia Solid-State Fermentation, Preparation and Detection of PESF

The *P. eryngii* strain ACCC 52700 (http://www.accc.org.cn) was inoculated in the PDA medium for 7 days at 25 °C before fermentation. The solid-state fermentation medium recipe was a mixture of corn-cob (80%), wheat bran (19%), lime powder (1%) with water packed in 500 mL transparent cylindrical glass bottles that were capped with an air filter membrane, autoclaved at 121 °C for 20 min, then cooled to room temperature, inoculated with *P. eryngii* strain under aseptic conditions, and sealed with a cap. Next, these bottles were moved to the artificial climate incubator in darkness, with environmental humidity set at 60% and temperature set at 25 °C for 14 days. as described in Supporting Information (Figure S1). The PESF was extracted with hot water (90 °C) incubation for 3 h and following procedures were referred as described previously [4,5].

The polysaccharide content was measured by the phenol-sulfuric acid method using glucose as a standard. The full wavelength scanning of the PESF sample (5 mg/mL) was scanned between 200 and 700 nm at intervals of 0.5 nm by a spectrophotometer (SpectraMax M2, Molecular Devices, San Jose, CA, USA). The polysaccharide components (5 mg/mL) were detected with high-performance liquid chromatography (HPLC) (1260 Infinity II, Agilent, Santa Clara, CA, USA) with differential detection by a carbohydrates analysis column (300 mm× 7.8 mm, Aminex HPX-87H) (Bio-Rad, USA) by 5 mM H_2_SO_4_ at 0.5 mL/min [3].

### 2.3. Cell Culture, Cell Oil Red O (ORO) Staining, MTT Assay

Macrophages (RAW 264.7 cells, ATCC TIB-71) were cultured in DMEM (glutamine, high glucose) supplemented with FBS (10%), penicillin (100 U/mL), and streptomycin (100 mg/mL) at 37 °C in 5% CO_2_. The cells were cultured for 12 h and incubated with 80 µg/mL oxLDL per mL of medium for 12 h. Then, the cells were divided into three groups, the oxLDL group, the oxLDL + PESF group and the normal group. Each group included cultured cells on three six-well plates for three independent experiments. During the next 24 h, the oxLDL-PESF group was exposed to PESF at a final concentration of 200 µg/mL in the medium, and the oxLDL group was added to the same amount of volume of medium as PESF. In the normal group, no oxLDL was added to the medium during the culture time [3].

Finally, cells in all groups were fixed in 4% paraformaldehyde for 30 min and stained by 0.5% oil red O (ORO) for 1 h. After removal of the floating colour by 60% isopropyl alcohol, an image of the cells was taken by a dissecting stereomicroscope (Olympus Co., Tokyo, Japan), showing the red lipid droplets in the cells stained by ORO. 

The quantification data of stainable lipid content correlated to microphotographs were normalized to an integral optical density (IOD) value by Image-Pro Plus (IPP) analysis software, which reflects lipid concentrations [15]. Data are expressed as the mean ± SD from three independent experiments. The significance is set at the levels * *p* < 0.05 and ** *p* < 0.01 compared with the control group. All treatments were carried out in triplicate.

The normalized degree of lipid accumulation (%) = (IOD drug treatment − IOD normal)/(IOD HCD − IOD normal).

The MTT assay was carried out as described previously [16].

### 2.4. Zebrafish Handling, ORO Staining and Intensity Quantification 

Adult zebrafish AB strains were obtained from Nanjing YSY Biotech Company Ltd. (Nanjing, China) and were housed in the light- and temperature-controlled aquaculture facility with a standard 14:10 h light/dark cycle and fed with live brine shrimp larvae bait twice daily at 28 °C in fish water (0.2% instant ocean salt in deionized water, pH 6.9–7.2, conductivity 480–510 mS·cm^−1^ and hardness 53.7–71.6 mg/L CaCO_3_). Four to five pairs of strong adult zebrafishes were chosen for natural mating every time and approximately 200–300 embryos were generated on average, which were hatched at 28 °C in clean fish water [14]. Zebrafish larvae at 6 days post-fertilization (d.p.f.), were chosen as an optimal stage and fed with 1% egg yolk power as the high-cholesterol diet (HCD) for the hyperlipidemia model development. Zebrafishes at 6 d.p.f. were placed in a culture dish with a 3 cm diameter at a density of 15 zebrafishes in 3 mL of fish water per group for treatment, and HCD was added to the dishes by a dilution at 1:100 (*v*/*v*) for a period of time. Finally, zebrafish larvae were fixed by formaldehyde (4%) for 1 h and then dehydrated by 25%, 50%, 75%, and 50% gradient methanol successively, followed by staining with 0.5% ORO for 24 h. Rehydration was achieved with 100%, 75%, 50%, and 25% gradient methanol and PBS in sequence. After ORO labelling, the lipids were easily visualized in tissues of the blood vessel, liver and gut under a dissecting stereomicroscope (Olympus Co., Tokyo, Japan). The quantification data of stainable lipid content correlated to microphotographs were normalized to an integral optical density (IOD) value as described in above Section 2.3, which reflects triacylglycerol and cholesterol concentrations.

The animal experimental protocol was approved by the Animal Care and Use Committee of Nanjing Normal University, China (permit no. 2090658) according to the governmental guidelines for animal care.

## 3. Results and Discussions

### 3.1. Extraction and Detection of Polysaccharides

**Figure 1:**
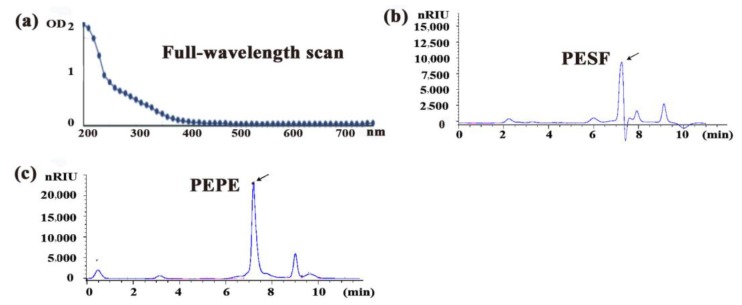
The full-wavelength scanning diagram from 200 to 700 nm (**a**) and the differential detection map of high-performance liquid chromatography (**b**) for the *P. eryngii* polysaccharide from mycelia solid-state fermentation (MSSF), using polysaccharide from fruiting bodies (PEPE) as a control (**c**).

The water-soluble polysaccharide of *P. eryngii* from the mycelia solid-state fermentation (MSSF) (Figure S1) was extracted by hot water and about 3.32% polysaccharides verses to the initial dried raw medium weight (g/g) were obtained, referred as PESF. The purity and the component of PESF was analysed by a full-wavelength scanning and HPLC respectively, using polysaccharides from *P. eryngii* fruiting bodies (PEPE) as control (Figure 1).

As shown in Figure 1a, there was no detectable absorbance peak at 260 or 280 nm for PESF, suggesting that nucleic acids or proteins did not exist in PESF. Furthermore, the total polysaccharide content of the PESF product was 82% (g/g). 

HPLC chromatograms showed that the main fraction of PESF appeared at approximately 7.3 min of retention time (Figure 1b), is consistent with that of PEPE (Figure 1c) whose lipid-lowering effect was tested and verified in high-fat-loaded mice in a previous study [4]. The results suggest that the *P. eryngii* polysaccharide PESF from MSSF may also has similar functions to PEPE from the fruiting body in potential lipid-lowering effects.

### 3.2. Safety Assessment and Lipid-Lowering Effect of PESF in a Macrophage-Derived Foam Cell Model

**Figure 2:**
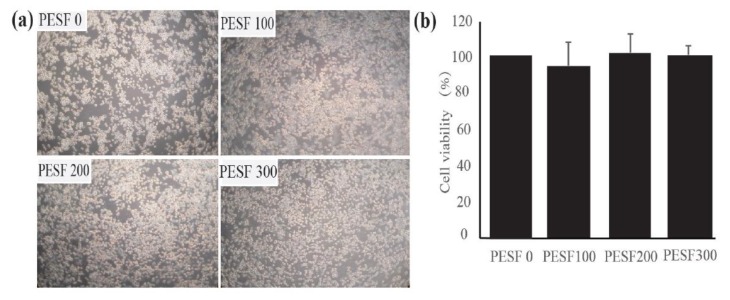

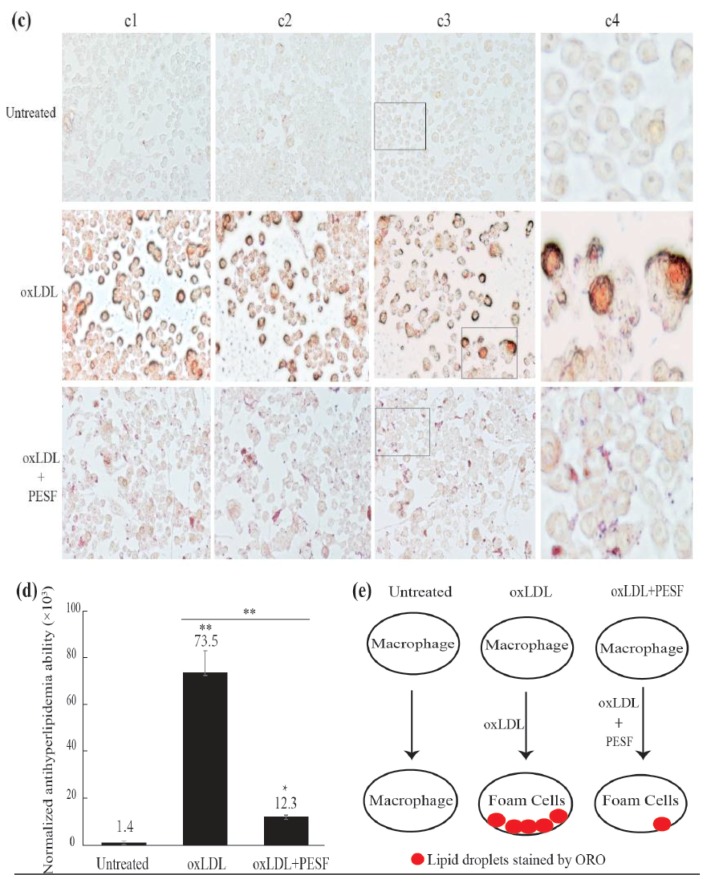
The *P. eryngii* polysaccharide from solid-state fermentation (PESF) was able to reduce accumulation induced by oxidized low density lipoprotein (oxLDL) in murine macrophages (RAW264.7 cells) with no detectable toxicity on cell viability. (**a**) Microphotographs of cells co-cultured with different concentrations of PESF (0, 100, 200, and 300 µg/mL) for 24 h by a phase contrast microscope. (**b**) Quantification data of the MTT assay showed the viability of cells affected by different concentrations of PESF. (**c**) Microphotographs of cells in the the oxLDL group, the oxLDL + PESF group and the normal group, showed intracellular red lipid droplets stained by oil red O (ORO) (a1, a2, a3). The photographs a4 were amplified with the box part of a3. (**d**) The quantification data of stainable lipid content correlated to microphotographs (**c**). (**e**) Overview of the experiment in murine macrophage-derived foam cells. The macrophages are induced by oxLDL to form foam cells, resulting in the excessive accumulation of red lipid droplets stained by ORO inside the cells of the oxLDL group. PESF inhibited lipid accumulation in the formation of foam cells in the PESF group, using normal macrophages as control.

To test the safety of PESF, the murine macrophages (RAW264.7 cells) were co-cultured with different final concentrations of PESF (0, 100, 200, and 300 µg/mL) for 24 h. Then, micrographs of the cultured cells were taken by a phase contrast microscope (Figure 2a) and a cell viability assay (MTT assay) was performed (Figure 2b).

The assay indicated that there were no significant differences in either the cell number or viability between PESF groups with different concentrations and the control, suggesting that PESF has no detectable toxicity on the cell viability in RAW264.7 cells.

To identify the lipid-lowering effect of PESF, we used macrophage-derived foam cells as a high-fat model. Murine RAW 264.7 macrophages were cultured and induced by oxLDL and divided into three groups, the oxLDL, the oxLDL + PESF and the normal groups. As shown in Figure 2c,d, macrophages-formed foam cells had been successfully induced by oxLDL, resulting in an excessive accumulation of stainable lipid droplets inside the cells of the oxLDL group. In comparison, there were no accumulated stainable lipid droplets in the normal group without treatment of oxLDL induction. As predicted, the intracellular lipid accumulation showed statistically significant decreases (*p* < 0.01) in the oxLDL + PESF group treated by PESF (200 µg/mL) for 24 h, resulting in only 16.73% of lipid content left inside the cell compared to 100% in the oxLDL group. 

These data suggest that PESF has biological functions in the inhibition of lipid accumulation in the formation of foam cells. Therefore, PESF was selected to be further studied in vivo in the animal model.

### 3.3. Establishment of the Hyperlipidemia Zebrafish Model

**Figure 3:**
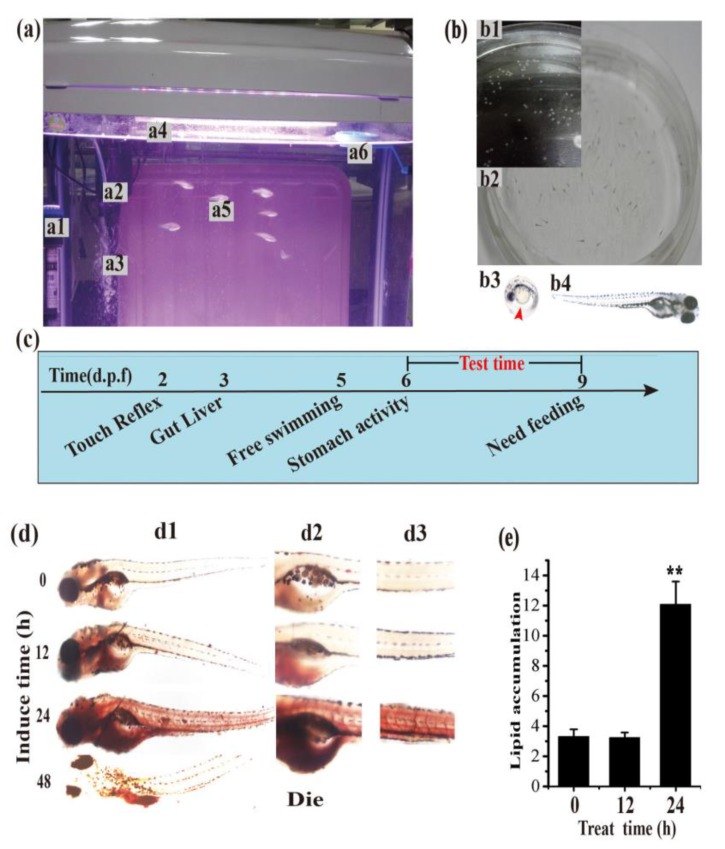
Establishment of the zebrafish larvae hyperlipidemia model. (**a**) Photographs of zebrafish during breeding. Adult zebrafish (a5) were fed with live brine shrimp twice daily and housed in a light- and temperature-controlled aquaculture facility (a) installed with a thermometer (a1), heating rods (a1), a filter device (a2), an oxygen pump (a3), lamp (a4), and a feeding ring (a6) with a standard 14:10 h light/dark cycle at 28 °C in fish water. Four to five pairs of adult zebrafish were set up for natural mating every time; (**b**) Representative photographs of zebrafish embryos and larvae. On average, 200–300 embryos (b1) are generated. Embryos and larvae (b2) were maintained at 28 °C in fish water on the open plate in the light of the incubator. Microphotographs showing that the zebrafish embryos (b3) and larvae (b4) are transparent all the way around except for little black scales on the body surface by a dissecting stereo microscope; (**c**) The developmental introduction of zebrafish larvae. Zebrafish larvae of 6 days post-fertilization (d.p.f.) begin to show stomach activity and can live without food supplementation within 9 d.p.f. Thus, zebrafish larvae at 6 d.p.f. were selected for the experiment model in the next experiment; (**d**) Representative microphotographs of the whole body (d1), the gut (d2) and the blood vessels (d3) with red stainable lipids induced by the high-cholesterol diet (HCD) at different times (0, 12, 24, 48 h) in the zebrafish larvae hyperlipidemia model by a dissecting stereomicroscope. Stainable lipids in the zebrafish larvae increased uniformly and suddenly with feeding time from 12 to 24 h and caused death at 48 h; (**e**) Normalized quantification data of stainable red lipid content correlated to microphotographs (**d**) in the zebrafish hyperlipidemia model.

To further identify the hypolipidemic effect of PESF in vivo, we selected zebrafish larvae as an animal model for the screening of hypolipidemic compounds and efficacy assessments, because zebrafish larvae have advantages of transparency, high reproduction capacity, and easy breeding (Figure 3). The zebrafish larvae (Figure 3(b4)) are transparent and very suitable for in vivo staining and microscopic observation. With the development of organs, larvae at 6 days post-fertilization (d.p.f.) have stomach activity and larvae do not need food supplementation until 9 d.p.f. Therefore, the zebrafish larvae between 6 and 9 d.p.f. were chosen for followed experiments (Figure 3c).

To develop a zebrafish larvae hyperlipidemia model, a suitable treatment time-schedule for hyperlipidemia induced by fatty food had been set up firstly. Zebrafish larvae at 6 d.p.f were fed with the high-cholesterol diet (HCD) for 0, 12, 24 and 48 h separately, and then stained by ORO in vivo to visualize the lipid content in the whole body, the gut and the blood vessels with a dissecting stereomicroscope.

As shown in Figure 3d, compared with limited stainable body lipids of red color in the zebrafish larvae at 0 h of HCD feeding, the body lipids increased slightly from 12 to 24 h, during which period of time the lipids engorged uniformly and suddenly in the whole body, especially into the gut and the blood vessels, resulting in about 3.65 times more lipids than that of 0 h. Notably, all zebrafish larvae feed by HCD for 48 h were died, suggesting a long-time HCD-overloading is very toxic and even lethal for larvae. 

The increased level of ORO staining indicates that lipids in the zebrafish larvae gut and blood vessels increased along with treatment time within 24 h, which is consistent with a report indicating that a time-dependent increasing in whole-larval triacylglycerol content. The results suggest that the zebrafish larvae hyperlipidemia model can be created with zebrafish of 6 d.p.f. by feeding HCD for 24 h. The lipid accumulation in the zebrafish gut and blood vessels occurred rapidly during the HCD feeding time from 12 to 24 h; therefore, this period would be selected as the treatment time-point for the assessment of hypolipidemic compounds in our next experiments. 

### 3.4. Lipid-Lowering Effect of PESF in a Zebrafish Larvae Model 

**Figure 4:**
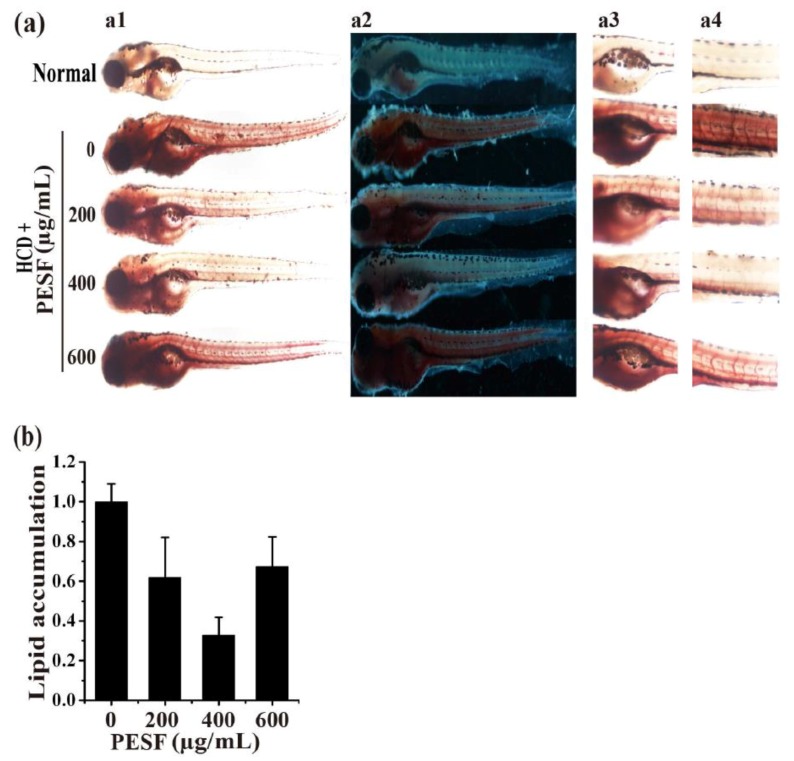
Lipid-lowering effect of PESF in the zebrafish hyperlipidemia model. (**a**) Representative photographs of zebrafish larval appearance with stainable lipids treated by PESF at different concentrations of 0, 200, 400 and 600 µg/mL respectively, in light (a1) and dark (a2) fields by a dissecting stereomicroscope. Stainable lipids in the zebrafish whole body (a1) (a2), gut (a3) and blood vessels (a4) decreased with PESF at concentrations of 0 to 400 µg/mL, but not 600 µg/mL. (**b**) Lipid content was normalized to the integral optical density (IOD) data through gut and blood vessel (a1) in the zebrafish larvae hyperlipidemia model.

To identify the lipid-lowering effect of PESF in vivo, we used the zebrafish larvae model established as described in Section 3.3 above. The zebrafishes at 6 d.p.f were fed with HCD for 24 h and exposed to PESF at different final concentrations of 0, 200, 400 and 600 µg/mL for 15 fishes in 3 mL of fish water during 12 to 24 h, and the body lipids increased slightly during this period (Figure 3). Then, they were stained by ORO at 24 h for image acquisition (Figure 4a), followed by quantitative image analysis of stainable lipid content in the gut and blood vessels correlated to microphotographs in the zebrafish larvae hyperlipidemia model. For the normal group, no HCD was fed during the culture time. 

As expected, PESF was able to significantly reduce the amount of lipid droplets in the zebrafish larvae hyperlipidemia model. At concentrations of 200, 400 and 600 µg/mL, PESF decreased the lipid content to 62% ± 20%, 33% ± 9%, and 67% ± 15% respectively, compared to 100% in the PESF group at a concentration of 0 µg/mL as the control (Figure 4b). Among these treatments, the most effect on zebrafish hyperlipidemia was observed for PESF at the concentration of 400 µg/mL. The results suggest that PESF has bioactivity in the inhibition of lipid accumulation in this zebrafish larvae hyperlipidemia model. 

### 3.5. Lipid-Lowering Comparison between PESF and a Commercial Hypolipidemic Drug-Simvastatin 

**Figure 5:**
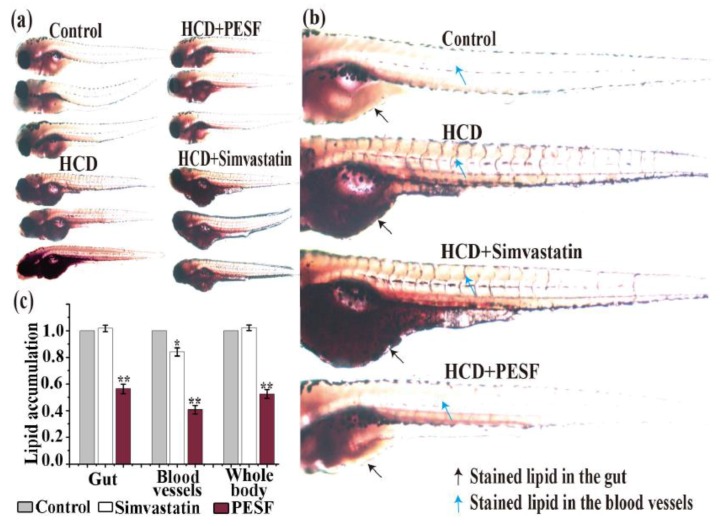
Comparison of PESF and simvastatin on inhibition of lipid accumulation in the zebrafish larvae model. (**a**) Representative photographs of zebrafish larval appearance with stainable lipids treated by PESF and simvastatin in the zebrafish model. Zebrafishes at 6 d.p.f were fed with HCD from 0 h to 24 h uninterruptedly and exposed to PESF (200 µg/mL) and simvastatin (200 µg/mL) from 12 h to 24 h and then stained by ORO at 24 h for image acquisition. For the zebrafishes in the normal group, no HCD was fed during the culture time. Conversely, the HCD group was fed with HCD for 24 h uninterruptedly. (**b**) Representative photographs of zebrafish larval appearance with stainable red lipids treated by PESF and simvastatin compared with the HCD group and the normal group. Black arrows point to lipids in blood vessels and blue arrows point to lipids in the gut. (**c**) Normalized quantification data of stainable lipid content in the zebrafish (gut, blood vessels, whole body) treated by PESF and simvastatin, correlated to microphotographs (a1), based on the HCD group set as 100%.

To further determine whether the zebrafish response to PESF is similar to clinical drugs in the zebrafish larvae hyperlipidemia model, simvastatin was selected as the comparative agent. Simvastatin is a well-known human hypolipidemic drug that inhibits cholesterol biosynthesis to prevent atherosclerosis-related complications such as stroke and heart attacks. With the method described above, we tested the lipid-lowering effect of PESF and simvastatin at the concentration of 200 µg/mL for the zebrafish larvae hyperlipidemia model, using normal zebrafish group and the HCD zebrafish group as controls (Figure 5). 

The lipid-lowering effect of PESF and simvastatin showed differences in this zebrafish larvae hyperlipidemia model. As shown in Figure 5c, based on the lipid accumulation level of the HCD group set as 100%, the levels of lipid accumulation in the HCD + simvastatin and HCD + PESF group were respectively 101.7% ± 23.4% and 56.3% ± 35.7% in the gut, 84.0% ± 30.7% and 40.6% ± 31.1% in the blood vessels, and 102.2% ± 20.2% and 52.4% ± 33.0% in the whole body. Stainable lipids in the zebrafish whole body decreased in the HCD + PESF group, but not in the HCD + simvastatin group, compared with the HCD group and the normal group. These data indicated that PESF significantly reduced the lipid accumulation levels both in the gut and the blood vessels. However, simvastatin did not reduce the lipid accumulation levels in the gut at all, and only reduce slightly in the blood vessels. The results indicated that PESF has a different mechanism to inhibit lipid accumulation with simvastatin that involves the suppression of hydroxy methylglutaryl coenzyme A (HMG-CoA) reductase to control cholesterol biosynthesis in the liver [17].

## 4. Conclusions

*P. eryngii* polysaccharides produced from mycelia solid-state fermentation (PESF) with strain ACCC 52700 using corn-cob and wheat bran as cultural materials, has similar components and functions to that from fruiting bodies. PESF could significantly inhibit lipid accumulation in both the macrophage-derived foam cell and zebrafish larvae hyperlipidemia models and behaved mainly through the lipid absorption process. We developed a zebrafish larvae hyperlipidemia model with the advantages of a short duration requirement for an in vivo efficacy assessment and research on lipid-lowering food additives or drugs. This study has developed a novel production model for lipid-lowering polysaccharides under mycelia solid-state fermentation with edible fungi from lignocellulolitic wastes in an economical and effective way.

## Supplementary Materials

The following are available online at http://www.mdpi.com/2073-4360/10/5/492/s1.

## Abbreviations

d.p.f.	days post-fertilization
DMEM	Dulbecco’s modified eagle medium
HCD	high-cholesterol diet
MSSF	mycelia solid-state fermentation
ORO	oil red O
oxLDL	oxidized low density lipoprotein
OD	optical density
PEPE	polysaccharides from *P. eryngii* fruiting bodies
PESF	polysaccharides from *P. eryngii* mycelia solid-state fermentation
SD	standard deviation
