# Supplementary material for: Lipid-Lowering Effect of the Pleurotus eryngii (King Oyster Mushroom) Polysaccharide from Solid-State Fermentation on Both Macrophage-Derived Foam Cells and Zebrafish Models
Source: Polymers (Basel). 2018 May 3;10(5):492. doi: 10.3390/polym10050492 (PMC6415515; doi:10.3390/polym10050492)
Supplement: Supplementary file 1 [file polymers-10-00492-s001.pdf]

Supporting Information

S1. Evaluation of production conditions for *P. eryngii* MSSF

To optimize the initial moisture of the mixture medium (g water / g wet basis) for *P. eryngii* mycelia in solid-state fermentation (MSSF), six groups of the material mixture were set up in glass tubes containing 10 g of the material mixture (80% corn-cob, 19% wheat bran and 1% lime powder) with different initial moistures of 40%, 50%, 60%, 70%, 80%, and 90% (g water / g wet basis) in this experiment, respectively. The appearance properties of fermented *P. eryngii* mycelia were checked after being cultured at 25 °C for 14 d (Fig. S1a).

Fig.S1

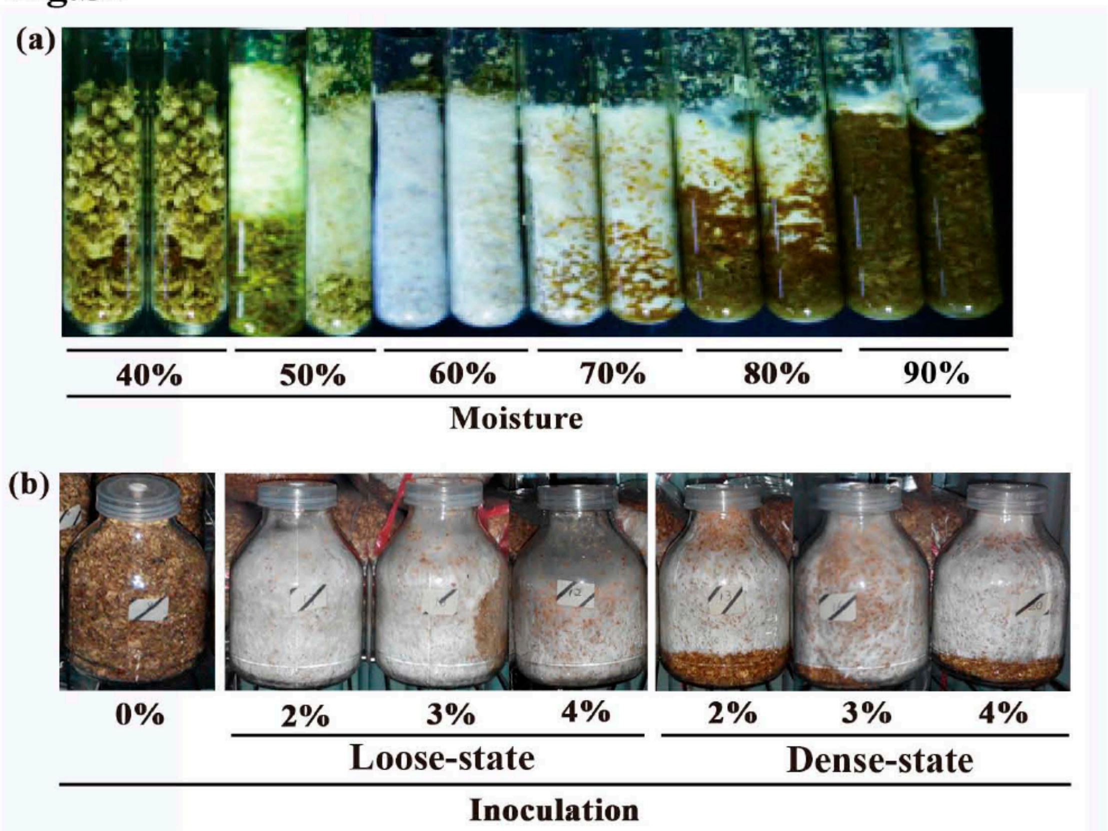

As shown in Fig. S1a, according to the white colour of the fermented *P. eryngii* mycelia covering the solid-state medium, the fermented mycelia in the group with 60% moisture had both the largest coverage and the highest mycelia density among all of groups, followed by the group with 70% moisture. In comparison, the coverage of fermented mycelia became smaller with lower and higher moisture contents below

50% or beyond 60%. It indicated that the optimum initial moisture of the mixture medium was 60% for the production of *P. eryngii* mycelia biomass by MSSF, meaning that the optimum ratio of dry material to water is 1:1.4. Furthermore, to optimize the package condition for the medium quantity in each cultural bottle for *P. eryngii* MSSF, two groups were set up with 150 g (loose-state) and 200 g (dense-state) of media, respectively in 500 mL bottles under the optimum initial moisture of 60%. Fermented mycelia were checked after having cultured at 25°C for 14 d, using solid-state medium as a control (Fig. S1b).

According to the white colour of the fermented mycelia covering the solid-state medium as shown in Fig. S1b, the mycelia covered the media completely in all of the loose-state groups, however, the mycelia did not cover the media at the bottom of the bottle in the dense-state groups. The results indicate that *P. eryngii* MSSF with 150 g (loose-state) medium in 500 mL bottles under an initial moisture of 60% is more effective than that with 200 g (dense-state). This may suggest the *P. eryngii* MSSF demands much more dissolved oxygen to produce fermented mycelia.
